# Kikuchi Fujimoto disease associated with cryptogenic organizing pneumonia: case report and literature review

**DOI:** 10.1186/1471-2334-10-64

**Published:** 2010-03-11

**Authors:** Feng Hua, Lei Zhu

**Affiliations:** 1 Department of Pulmonary Medicine, Zhongshan Hospital, Fudan University, 180 Feng Lin Road, Shanghai, 200032, PR China; 2 Department of Pulmonary Medicine, Huzhou central Hospital, 198 Hongqi Road, Huzhou city, Zhejiang province, 313000, PR China

## Abstract

**Background:**

The association of Kikuchi Fujimoto disease (KFD) with cryptogenic organizing pneumonia (COP) is extremely rare. We report a case of simultaneous diagnosis of KFD and COP.

**Case Presentation:**

A 33-year-old male presented with a 1-month cough illness and fever lasting for 5 days. The chest radiograph revealed double lower lobe infiltrate, which was unresponsive to antibiotics. A cervical lymph node was first found in the development of this disease. Bronchoscopy, bronchoalveolar lavage and lung biopsy established the diagnosis of COP, while a lymph node biopsy was consistent with KFD. The patient improved on steroids.

**Conclusions:**

KFD and COP are possible part of a disease continuum, rather than separate entities.

## Background

Cryptogenic organizing pneumonia (COP) is an interstitial lung disease characterized by intra-alveolar buds of connective tissue [1]. It can be idiopathic or associated with a known underlying disease.

In this case report, we present a 33-year-old man with COP, followed by concomitant Kikuchi Fujimoto disease (KFD). KFD was first described in 1972 by Kikuchi and Fujimoto and others. It is a clinicopathological entity that commonly presents with cervical lymphadenopathy and fever in the absence of an active infectious disease. KFD is a rare histiocytic necrotizing lymphadenitis, which has a benign self-limiting clinical course. Its origin is unknown, but an abnormal autoimmune reaction has been suggested and infection is often considered as an inducing feature [2].

To the best of our knowledge, this is the first case report of COP and KFD.

## Case Presentation

A 33-year-old man presented with a 1-month coughing illness and fever that lasted for 5 days. Before admission, the patient experienced expectoration of yellow sputum and antibiotic therapy for 2 weeks. There was no history of fever, rash or respiratory distress, and there was no family history of autoimmune or connective tissue disorders. Respiratory examinations revealed fine crackles in both lungs. Examination of other systems was unremarkable.

The laboratory investigation showed hemoglobin 140 g/L, leukocytes 3.9 × 10^9^/L with a normal differential count, platelets 142 × 10^9^/L, C-reactive protein 1.2 mg/L, and an erythrocyte sedimentation rate (ESR) of 14 mm/h. Heterophil agglutination trial was negative. Serum biochemistry findings including liver and renal function were within normal limits. The results of cellular immunity assay showed a decrease in the CD4/CD8 ratio (0.63). Test results were negative for toxoplasma, cytomegalovirus (CMV), rubella, Epstein-Barr virus (EBV), human herpes virus type 1 and human immunoddficiency virus (HIV). Also rheumatological and immunological tests were normal. These tests included antinuclear antibody, rheumatoid factor, antistreptolysin O, antineutrophil cytoplasmic antibody and the levels of serum C3 and C4 as well as IgG, IgA, IgE and IgM.

**Figure 1:**
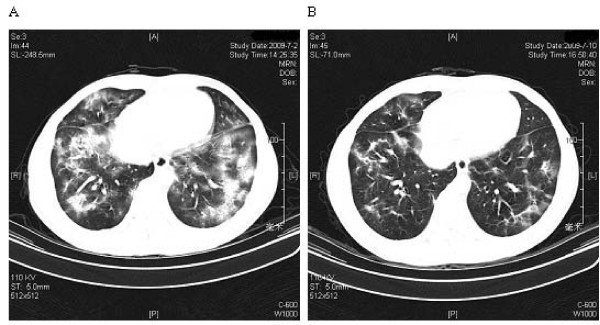
**Chest CT finding of COP**. A: HRCT was performed before glucocorticoids were administered, and showed patchy areas of ground-glass attenuation and consolidation in the lower lobes that contained air bronchograms. B: A follow-up HRCT was performed 8 days after glucocorticoid administration. It showed improvement in both the lungs with few residual lesions.

Bacteriological analysis of blood, sputum and urine did not reveal any pathogenic agents. Pulmonary function presented restrictive ventilatory functional disturbance. In addition, chest computed tomography (CT) demonstrated diffuse infiltrating lesions in both lungs, predominantly in the lower lobes (Fig. 1A).

With an initial diagnosis of bilateral pneumonia, moxifloxacin and cefepime were initiated. Eleven days after admission, a solitary lymph node was first found in the right anterior cervical chain. The lymph node was tender and measured 3 cm in diameter. At this point, the patients had leucopenia (4.3 × 109/L) and lymphopenia (0.8 × 109/L). The ESR was slightly accelerated to 40 mm/h. The serum C-reactive protein level was normal. The serum galactomannan enzyme immunoassay and 1, 3 β-glucan assays for diagnosis of invasive fungal infection were negative. Findings were negative for an intradermal tuberculin test, as well as for sputum and urine tests for the *Mycobacterium tuberculosis*. The serological test for coxsackie B virus suggested infection in the recent past.

**Figure 2:**
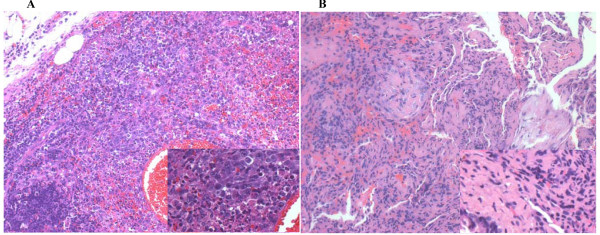
**Histopathological findings**. A: Lymph node histology of the patient showed aggregation of histiocytes and plasmacytoid monocytes with focal necrosis, scattered pyknotic nuclei and karyorrhexis (HE, ×20). Insert shows more details (HE, ×40). B: Lung biopsy illustrating intraluminal fibrous tissue plugs with adjacent thickened interstitium, intraalveolar macrophages, and lymphoplasmacytic inflammation (HE, ×20). Insert shows more details (HE, ×40).

A cervical lymph node biopsy showed normal lymph node architecture in some areas, but a few irregular necrotic areas with intense karyorrhexis. Around the necrotic areas, macrophages, plasma cells, histiocytes and lymphocytes were noted. No neutrophils were detected (Fig. 2A). No tuberculoid granuloma, caseating necrosis, or evidence of malignancy was found. Immunohistochemistry studies showed that the lymphoid cells adjacent to the necrotic foci were positive for CD3, LCA and CD45RO, but negative for CD20. CD79-positive cells were seen farther away from the necrotic foci. Histiocytic cells at the periphery of the necrotic foci were strongly positive for CD68. This histological pattern was suggestive of KFD [3].

Bronchoalveolar lavage fluid showed no increase in eosinophil or lymphocyte counts, but a decrease in the CD4/CD8 ratio (0.52). Lung biopsy by video-assisted thoracoscopy was performed 12 days post-admission and revealed granulation tissue in the alveolar spaces, with associated marked alveolar wall thickening and frequent metaplasia of bronchiolar and alveolar cells (Fig. 2B). The staining of reticular fiber, PAS and hexamethylenetetramine silver were negative. These findings of BAL and lung biopsy were compatible with COP [4,5].

After the diagnosis of KFD associated with COP, 45 mg/day prednisone was administered, with a gradual improvement in symptoms. A second HRCT was done, which showed marked resolution of the alveolar exudates (Fig. 1B), and cervical lymphadenopathy had resolved 8 days post-treatment. At 12 weeks follow-up, the patient remained well, with no other symptoms or lymph node enlargement.

## Conclusions

COP is a rare disease. The characteristic clinical features of COP are flu-like illness, followed by progressive dyspnea, patchy infiltrates on chest radiography and CT, a restrictive spirometric pattern with diffusion impairment. And, the histopathological pattern of COP is intraluminal organization, predominantly within the alveolar ducts and alveoli [6].

KFD was first described in 1972 by the Japanese pathologists Kikuchi and Fujimoto as a benign, self-limited syndrome of necrotizing lymphadenitis with a characteristic histopathological appearance that mimics malignant lymphoma. It typically presents as localized lymphadenopathy, predominantly in the cervical region, accompanied by fever and leukopenia in up to 50% of cases [7].

Reports in the literature of an association between respiratory manifestation and KFD are not frequent. In 1990, Tanaka et al. [8] reported one patient with KFD who presented with respiratory symptoms, and T-lymphocyte alveolitis was diagnosed. Additionally, antinuclear antibodies were positive, and antibodies against human T-lymphotrophic virus type 1 (HTLV-I) were false positive. These findings suggested an immunological abnormality. Cervical lymph node swelling and infiltrative shadow on chest X-ray film improved with steroid therapy.

Several suggestions on the etiology of COP or KFD have been raised. Certain microorganisms (EBV, HTLV-I, herpes human 6 virus, *Toxoplasma*, parvovirus B19, CMV, *Brucella*, *Yersinia enterocolitica *and parainfluenza virus) have been suggested as the causative agents of the disease, and initiate a hyper-immune response of the T cells and histiocytes to the infectious agents. Positive serological test for Coxsackie B virus in this case might be same mechanism. However, none of these possibilities have been definitively proven [9,10]. In addition, many cases of KFD in patients with lupus have been reported. KFD has also been reported in other autoimmune diseases, including Still disease [11], rheumatoid arthritis [12], polymyositis [13], and mixed connective tissue disease [14].

Similarly, COP has been described in various autoimmune diseases [15], primarily in rheumatoid arthritis [16] but also in systemic lupus erythematosus and polymyositis [12]. It is clear that an immunological abnormality is the common pathogenesis of COP and KFD. Although the precise mechanism may be different, response of corticosteroids on this case indicated that the pathogenesis of both diseases may be immune-mediated. Therefore, the concomitant appearance of COP and KFD probably reflects two diseases that are part of a continuum, rather than separate entities.

In summary, we presented a case of COP that was diagnosed concurrently with KFD. The potential etiology of concomitant COP and KFD in our case could have been a nonspecific reaction to lung injury, unclear immunologically mediated response, occult virus infection, or immunosuppression. Further studies on a series of COP patients are necessary to elucidate this mechanism.

## Consent

Written informed consent was obtained from the patient for publication of this case report and any accompanying images. A copy of the written consent is available for review by the Editor-in-Chief of this journal.

## Competing interests

The author denies that he has any intention to obtain any financial interests.

## Authors' contributions

FH has acquisition of data and interpretation of data; LZ has been involved in drafting the manuscript. Both authors read and approved the final manuscript.

## Pre-publication history

The pre-publication history for this paper can be accessed here:

http://www.biomedcentral.com/1471-2334/10/64/prepub
